# Oncogenic ALK regulates EMT in non-small cell lung carcinoma through repression of the epithelial splicing regulatory protein 1

**DOI:** 10.18632/oncotarget.8955

**Published:** 2016-04-23

**Authors:** Claudia Voena, Lydia M. Varesio, Liye Zhang, Matteo Menotti, Teresa Poggio, Elena Panizza, Qi Wang, Valerio G. Minero, Sharmila Fagoonee, Mara Compagno, Fiorella Altruda, Stefano Monti, Roberto Chiarle

**Affiliations:** ^1^ Department of Molecular Biotechnology and Health Sciences, University of Torino, Torino, Italy; ^2^ Center for Experimental Research and Medical Studies (CERMS), Città della Salute e della Scienza, Torino, Italy; ^3^ Department of Pathology, Children's Hospital and Harvard Medical School, Boston, USA; ^4^ Section of Computational Biomedicine, Boston University School of Medicine, Boston, USA; ^5^ Molecular Biotechnology Center, University of Torino, Torino, Italy

**Keywords:** lung cancer, ALK, EMT, ESRP1/2

## Abstract

A subset of Non-Small Cell Lung Carcinoma (NSCLC) carries chromosomal rearrangements involving the Anaplastic Lymphoma Kinase (ALK) gene. ALK-rearranged NSCLC are typically adenocarcinoma characterized by a solid signet-ring cell pattern that is frequently associated with a metastatic phenotype. Recent reports linked the presence of ALK rearrangement to an epithelial-mesenchymal transition (EMT) phenotype in NSCLC, but the extent and the mechanisms of an ALK-mediated EMT in ALK-rearranged NSCLC are largely unknown. We found that the ALK-rearranged H2228 and DFCI032, but not the H3122, cell lines displayed a mesenchymal phenotype. In these cell lines, oncogenic ALK activity dictated an EMT phenotype by directly suppressing E-cadherin and up-regulating vimentin expression, as well as expression of other genes involved in EMT. We found that the epithelial splicing regulatory protein 1 (ESRP1), a key regulator of the splicing switch during EMT, was repressed by EML4-ALK activity. The treatment of NSCLC cells with ALK tyrosine kinase inhibitors (TKIs) led to up-regulation of ESRP1 and E-cadherin, thus reverting the phenotype from mesenchymal to epithelial (MET). Consistently, ESRP1 knock-down impaired E-cadherin up-regulation upon ALK inhibition, whereas enforced expression of ESRP1 was sufficient to increase E-cadherin expression. These findings demonstrate an ALK oncogenic activity in the regulation of an EMT phenotype in a subset of NSCLC with potential implications for the biology of ALK-rearranged NSCLC in terms of metastatic propensity and resistance to therapy.

## INTRODUCTION

Approximately 5-6% of NSCLC cases harbor chromosomal rearrangements involving the Anaplastic Lymphoma Kinase (ALK) gene that produce fusion proteins with constitutive ALK kinase activity [1, 2]. The most frequent is the chromosomal inversion within chromosome 2 that generates EML4-ALK chimeric protein, but additional ALK translocations with different partners have been described [3–5]. ALK-rearranged NSCLC is typically an adenocarcinoma that exhibits unique histological features represented by solid tumor growth and frequent signet-ring cells with abundant intracellular mucin [2, 6–10]. In lung cancers, as well as other epithelial cancers, the signet ring morphology is frequently associated with a more aggressive and metastatic phenotype [11–14]. Interestingly, in NSCLC some studies reported a strong association of ALK rearrangements with advanced disease stage at diagnosis and metastasis [8, 10]. In addition, when compared to other NSCLC, ALK-rearranged NSCLC is frequently associated with an epithelial-mesenchymal transition (EMT) phenotype as determined by loss of E-cadherin and increased vimentin expression [9]. The EMT is a fundamental program activated during embryogenesis and development that converts a polarized-epithelial cell into a mesenchymal cell with higher motility capacity and elevated resistance to apoptosis [15–17]. In epithelial tumors, EMT is a crucial process for the dissemination of cancer cells and it has been related to progression and metastasis, and a mesenchymal-like phenotype is frequently observed in aggressive tumors [15, 16, 18–20]. In cancer cells, EMT is usually characterized by the suppression of epithelial markers, such as E-cadherin, and expression of mesenchymal markers, such as vimentin and N-cadherin through a complex transcriptional reprogramming [21–23]. EMT phenotype is also thought to be associated with resistance to targeted therapy in EGFR-driven NSCLC [24, 25] and in ALK-rearranged NSCLC treated with ALK TKIs [26] as well as in K-Ras mutated cancers [27]. Moreover, recent reports demonstrated that EMT was dispensable for metastasis but contributed to chemoresistance in two different mouse models [28, 29].

In this study we found that 2 out of the 3 most commonly used ALK-rearranged NSCLC cell lines show an EMT phenotype based on E-cadherin and vimentin expression. In these two cell lines, the inhibition of ALK kinase activity with ALK tyrosine kinase inhibitors (TKIs) or the knock-down of EML4-ALK by shRNA, reverted the mesenchymal-like phenotype leading to up-regulation of E-cadherin and down-regulation of vimentin, so-called mesenchymal to epithelial transition (MET) [21]. EML4-ALK repressed the epithelial splicing regulatory protein 1 and 2 (ESRP1 and ESRP2), key regulators of a splicing switch during EMT. Overexpression of ESRP1 in these ALK-rearranged NSCLC cell lines lead to up-regulation of E-cadherin. Consistently, ESRP1 knock-down impaired the reversion to an epithelial phenotype associated to inhibition of ALK activity. Thus, in ALK-rearranged NSCLC, the EMT phenotype depends on the ALK activity via ESRP repression.

## RESULTS

### EMT markers are enriched in ALK-rearranged NSCLC

Early reports suggest a possible role of ALK in inducing an EMT phenotype in ALK-negative lung cancer cells [30]. However, the real contribution of the ALK oncogene to an EMT phenotype has not been investigated in ALK-rearranged NSCLC. To determine the association of an EMT phenotype with the presence of ALK rearrangements in NSCLC, we analyzed data sets of primary ALK-rearranged NSCLC as well as ALK-rearranged human cell lines for EMT signatures and the EMT markers E-cadherin, vimentin and N-cadherin. We identified 16 ALK-rearranged NSCLC cases from publicly available datasets (TCGA and GEO dataset GSE31210) [31, 32] and compared them to 29 normal samples [33].

**Figure 1:**
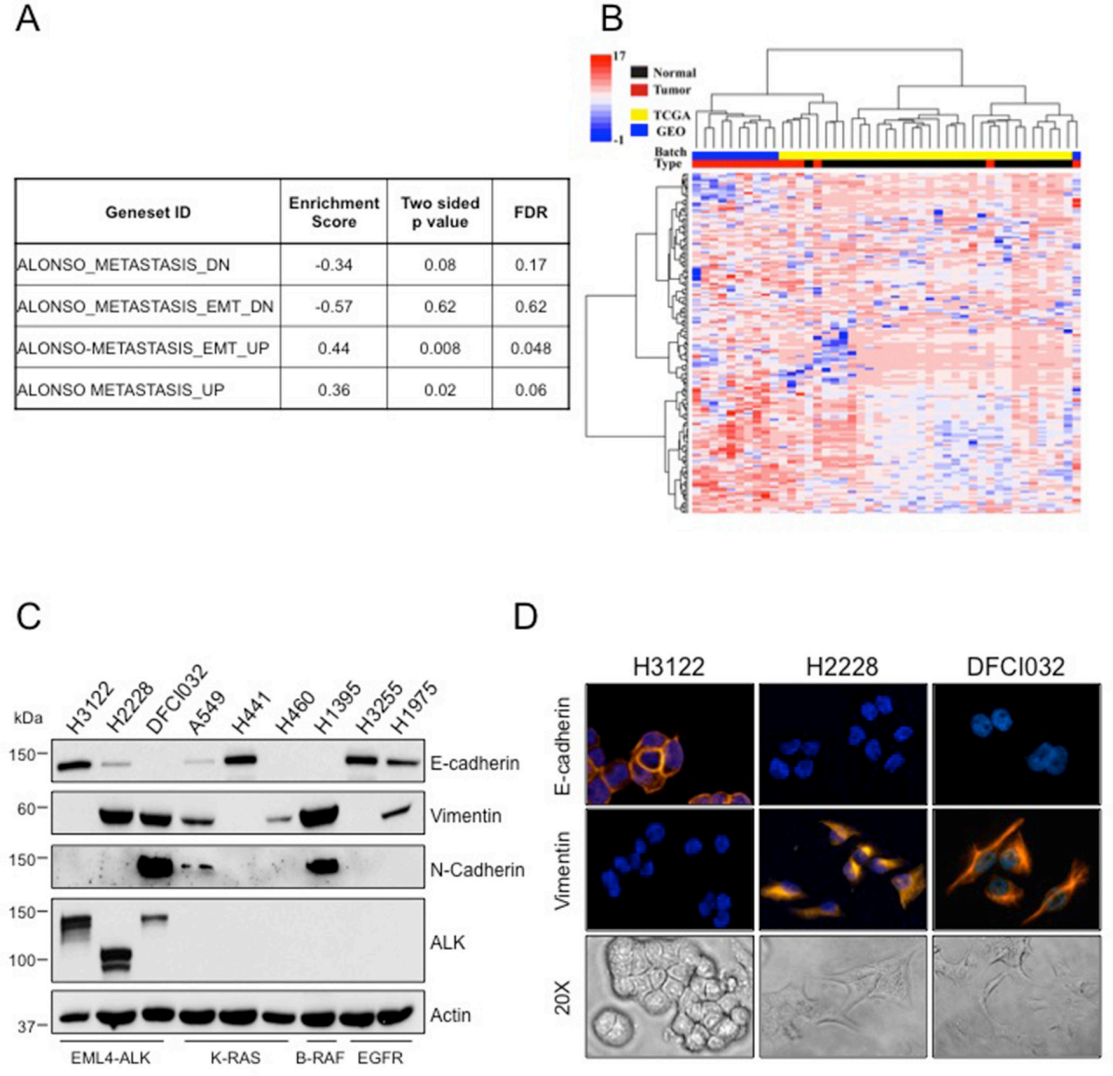
ALK-rearranged NSCLC are enriched in EMT markers **A.** The enrichment of EMT signatures of MSigDB on the differentially expressed genes between ALK-rearranged tumors and normal samples based on the KS test. **B.** A heatmap of clustered data (both on the gene and sample levels) is shown, selected data of the EMT activated signatures genes were included. Blue color indicates GEO batch and yellow color indicates TCGA batch. Red color indicates ALK positive tumors and black color indicates normal samples. **C.** Human NSCLC cell lines harboring different genetic lesions were blotted with the indicated antibodies. **D.** Immunofluorescence staining for E-cadherin and vimentin (top and central panels) and bright-field images showing morphology (bottom panels) of ALK-rearranged NSCLC cell lines, H3122, H2228 and DFCI032. Nuclei were stained with DAPI.

The list of genes differentially expressed between ALK-rearranged tumors and adjacent normal samples was tested for enrichment with respect to the EMT signatures in the MSigDB repository [34]. Gene Set Enrichment Analysis (GSEA) [35] yielded a statistically significant enrichment of the activated EMT signatures in ALK-rearranged NSCLC compared to control samples (Figure 1A). Concordantly, hierarchical clustering of the 45 samples projected onto the “ALONSO_METASTASIS_UP” signature yielded a clear segregation into ALK-rearranged tumors and normal samples (Figure 1B) [36]

Next, we examined the expression levels of the EMT markers, E-cadherin, vimentin and N-cadherin, in commonly available human NSCLC cell lines derived from different genotypes. ALK-rearranged NSCLC cell lines showed variable expression of EMT markers in two cells lines (H2228 and DFCI032), whereas a third commonly used cell line (H3122) was more epithelial. By Western Blot assay, H2228 and DFCI032 expressed high vimentin and little or no E-cadherin, whereas H3122 expressed only E-cadherin. DFCI032 also strongly expressed N-cadherin (Figure 1C). Consistently, H3122 displayed a classic epithelial morphology with expression of E-cadherin on the cell membrane, whereas H2228 and DFCI032 exhibited a mesenchymal phenotype with strong vimentin expression in the cytoplasm and almost undetectable E-cadherin (Figure 1D). NSCLC cell lines with other driver mutations (K-Ras, EGFR and B-Raf mutations) similarly showed variable expression of EMT markers (Figure 1C). Thus, heterogeneity of EMT phenotype was observed in ALK-rearranged NSCLC, consistent with the notion that a range of EMT marker expression is commonly observed within NSCLC [24, 27, 37].

### ALK oncogenic activity regulates EMT in ALK-rearranged NSCLC

**Figure 2:**
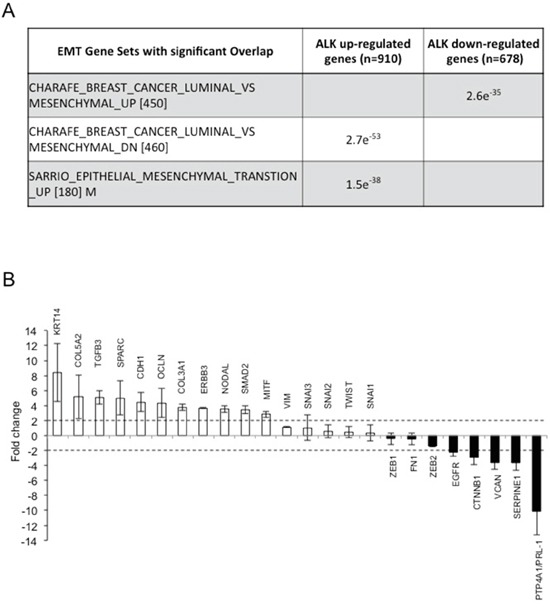
ALK oncogenic activity regulates EMT in ALK-rearranged NSCLC **A.** Top gene EMT related signatures of MSigDB CGP showing enrichment with the up-regulated and down-regulated genes of ALK based on hyper-geometric test. **B.** RT^2^ Profiler Array analysis of the H2228 cell line where EML4-ALK was inhibited for 24 hours with 300nM TAE-684 or crizotinib or knocked-down by shRNA for 72 hours. Histograms represent means of genes up- or down-regulated in all the three different treatments. Fold change levels are shown compared to controls (untreated cells). Dotted lines indicate upper or lower limits of significant changes.

We next investigated in detail whether EML4-ALK activity was directly controlling the EMT phenotype in those ALK-rearranged NSCLC with a mesenchymal phenotype. To this end, we focused our experiments on the H2228 and DFCI032 cell lines that showed an EMT phenotype (Figure 1C). We first performed an RNA sequencing (RNA-Seq) analysis on H2228 cells treated with the ALK TKI TAE-684 for 24 hours. We used Cuffdiff to identify differentially expressed genes at an FDR q-value ≤ 0.05 and found 910 genes whose expression decreased (ALK up-regulated genes) and 678 genes whose expression increased (ALK down-regulated genes) upon ALK inhibition (Supplementary Table 1) [38]. These sets of up- and down-regulated genes were then tested for enrichment against EMT gene sets derived from *in vitro* experiments and included in the MSigDB c2 CGP gene set compendium. We found that ALK activated or repressed genes significantly correlated with an EMT phenotype (Figure 2A), thus suggesting that ALK activity might directly regulate an EMT phenotype in ALK-rearranged NSCLC.

Next, we performed an RT^2^ Profiler PCR array containing 83 EMT-related genes on H2228 cells treated with two different ALK TKIs (TAE-684 and crizotinib) or where EML4-ALK was knocked-down by a specific shRNA (Supplementary Table 2). To exclude genes modulated by off-target activity of the TKI or the shRNA approach, we considered only genes that were consistently regulated in all the three different conditions. Upon ALK inhibition PTP4A1 (also known as PRL-1), SerpinE1 and CTNNB1, all genes that are associated with a mesenchymal or invasive phenotype [39–41], were strongly down-regulated. In contrast, E-cadherin (CDH1), occludin (OCLN) and keratin14 (KRT14) [21, 22, 42], all genes typically associated with an epithelial morphology, were markedly up-regulated (Figure 2B).

**Figure 3:**
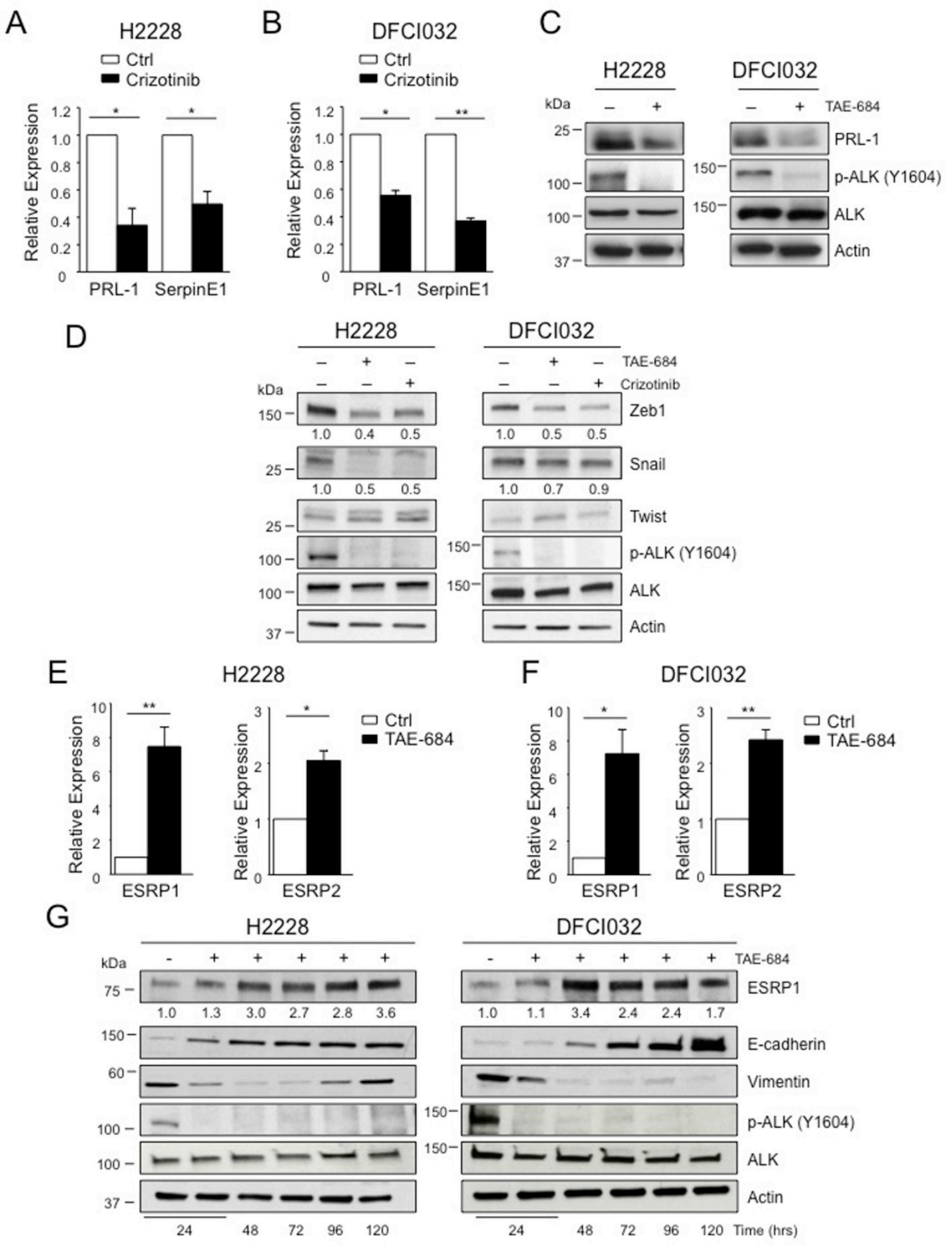
EML4-ALK regulates ESRP1 and ESRP2 **A-B.** H2228 (A) and DFCI032 (B) were treated with crizotinib (300nM) for 24 hours and collected for qRT-PCR analysis to check mRNA expression of PRL-1 and SerpineE1. **C.** H2228 and the DFCI032 cell lines were treated with TAE-684 (300nM) for 24 hours. Total cell lysates were blotted with the indicated antibodies. **D.** H2228 and DFCI032 cell lines were treated with TAE-684 or crizotinib (300nM) for 48 hours and the collected for Western blot analysis. Total cell lysates were blotted with the indicated antibodies. **E-F.** H2228 (E) and DFCI032 (F) were treated with TAE-684 (150nM) and collected at 96h for qRT-PCR analysis to check mRNA expression of ESRP1 and ESRP2. One representative experiment out of two is shown. **G.** H2228 and DFCI032 cell lines were treated with 300nM TAE-684 for the indicated time. Cells were collected and blotted with the indicated antibodies. Two-tailed Student's t tests were used to calculate the p values shown. Data are represented as mean (±SEM). *, *P*<0.05; **, *P*<0.005.

We validated some of the genes found in these screenings by quantitative RT-PCR (qRT-PCR) in both H2228 and DFCI032 cell lines. mRNA levels of PRL-1 and SerpinE1 showed significant changes in expression upon ALK inhibition in both cell lines (Figure 3A-3B), confirming the screening results. In keeping with the mRNA data, the protein expression levels of PRL-1 decreased and were dependent on ALK kinase activity (Figure 3C). Interestingly, one of the genes identified in the screening with the RT^2^ Profiler PCR array was ERBB3 that was strongly up-regulated after ALK inhibition both as mRNA (Figure 2B) and protein (Supplementary Figure 1A), consistent with our previous findings [43].

Based on these screenings, classical transcriptional regulators of EMT [21, 22], such as Snail, Twist or Zeb families, did not show any significant changes at the mRNA level (Figure 2B). Western blot analysis revealed down-regulation of Zeb1 protein in both cell lines, and Snail protein only in H2228 after treatment with ALK TKIs (Figure 3D), suggesting a post-transcriptional control of these transcription factors by oncogenic ALK. Some EMT regulators have been previously associated with ALK oncogenic activity [30], therefore we further extended our analysis to other EMT-related genes that were not included in the array. We found that ALK regulated the expression of ESRP1 and ESRP2, key regulators of a splicing switch during EMT [44, 45]. Interestingly, both ESRP1 and 2 were repressed in H2228 and DFCI032 cell lines and upon ALK inhibition their mRNA levels increased significantly (Figure 3E-3F and Supplementary Figure 1B-1C). Consistently, protein levels of ESRP1 were up-regulated by ALK inhibition (Figure 3G and Supplementary 1D). Up-regulation of ESRP1 mRNA and protein was also observed in H2228 cells transduced with shALK, thus excluding off-target effects of ALK TKIs on ESRP1 regulation (Supplementary Figure E-F), Concomitantly, both E-cadherin and vimentin were up- and down-regulated, respectively (Figure 3G and Supplementary Figure D-F). *In vivo* treatment with TAE-684 resulted in an increased staining for ESRP1 in s.c. xenografts of H2228 (Supplementary Figure 2A). Interestingly, in a human sample of our collection where we had ALK-rearranged NSCLC and adjacent normal lung, we detected lower staining of ESRP1 protein in tumor cells than in the adjacent normal epithelial cells (Supplementary Figure 2B). Taken together these results suggest that oncogenic EML4-ALK activity orchestrates a transcriptional and post-transcriptional program to sustain the EMT phenotype in ALK-rearranged NSCLC.

### EML4-ALK regulates E-cadherin and vimentin in ALK-rearranged NSCLC

**Figure 4:**
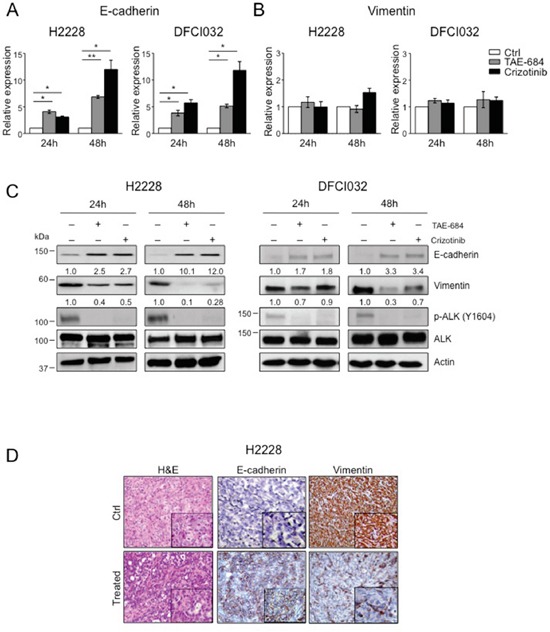
ALK oncogenic activity sustains the mesenchymal phenotype in ALK-rearranged NSCLC **A-B.** Real-time PCR analysis of mRNA levels of E-cadherin (A) and vimentin (B) in H2228 and DCI032 cells treated with TAE-684 or crizotinib (300nM) for the indicated time. mRNA expression values are calculated relative to controls. One representative experiment out of three is shown. **C.** H2228 and DFCI032 cells were treated with 300nM TAE-684 or crizotinib for the indicated time. Total cell lysates were blotted with the indicated antibodies. **D.** Representative hematoxilin-eosin (H&E) (left panels) and immunostaining with anti-E-cadherin (central panels) and anti-vimentin (right panels) antibodies on tumor xenograft sections from control (Ctrl) and mice treated with TAE-684. Data are from two independent experiments. Two-tailed Student's t tests were used to calculate the p values shown. Data are represented as mean (±SEM). For E-cadherin, *, *P*<0.05; **, *P*<0.005. For vimentin the p value was not significant.

Next, we determined to what extent the ALK oncogenic activity controlled the expression of EMT markers, E-cadherin and vimentin, in ALK-rearranged NSCLC. Based on the RNA-Seq results and the RT^2^ Profiler PCR array, EML4-ALK regulated only E-cadherin at transcriptional level, whereas mRNA levels of vimentin did not change upon ALK inhibition (Figure 2B). Thus, we performed a qRT-PCR analysis to study the mRNA levels of E-cadherin and vimentin in H2228 and DFCI032 treated with ALK TKIs. Upon treatment, E-cadherin mRNA levels significantly increased in both cell lines confirming that E-cadherin was transcriptionally regulated by ALK activity (Figure 4A). In contrast, no significant changes of vimentin mRNA levels in either cell line were detected, in keeping with the array data (Figure 4B). These data are also in accordance with Guo et al. that showed no changes in vimentin mRNA upon EML4-ALK ectopic expression in H1299 NSCLC cancer cell line [30]. Protein levels of E-cadherin and vimentin markedly changed when EML4-ALK was inhibited by TKIs in both H2228 and DFCI032 (Figure 4C). In particular, ALK inhibition led to up-regulation of E-cadherin and down-regulation of vimentin, thus reverting the phenotype from mesenchymal to epithelial in both cell lines (so called mesenchymal-to-epithelial transition, MET). We also treated the other ALK-rearranged NSCLC cell line characterized by an epithelial phenotype (H3122) to check whether the EMT markers, E-cadherin and vimentin, changed upon ALK inhibition and found that the level of E-cadherin expression remained stable and vimentin still undetectable for the whole period of inhibition with TKIs (Supplementary 3A), thus indicating that in this cell line ALK activity was not regulating an EMT phenotype. The effects of ALK TKIs were linked to a specific inhibition of ALK activity and not to off-target effects for the following reasons: 1) we observed similar effects with two unrelated ALK TKIs (Figure 4C); 2) ALK TKIs did not modulate EMT markers in NSCLC cell lines driven by different oncogenic mutations, such as K-Ras or EGFR mutations (Supplementary Figure 3B-3C); and 3) genetic knock-down of EML4-ALK closely phenocopied the effect of ALK TKIs on E-cadherin and vimentin mRNA (Supplementary Figure 4A-4B) and protein levels (Supplementary Figure 4C). EMT regulation by oncogenic ALK was also consistently observed *in vivo* in s.c. xenografts of H2228 treated with an ALK TKI (Figure 4D). Overall, these data showed that *in vitro* and *in vivo* the EMT phenotype in ALK-rearranged NSCLC is directly sustained by the oncogenic activity of ALK.

During cancer progression the activation of an EMT program, and thus the acquisition of a mesenchymal phenotype, is correlated to increased migratory and invasive properties of cancer cells or to chemoresistance [15, 16, 28, 29]. The reversion of the mesenchymal phenotype by ALK inhibition impaired the migratory and invasive abilities of DFCI032 cell line (Supplementary Figure 5A-5B). In contrast, in H2228 cells treatment with TAE-684 increased cell migration and invasion, suggesting the activation of compensatory pathways to sustain or even increase this phenotype (Supplementary Figure 5A-5B). We and others previously demonstrated that the EGFR family members can induce resistance to ALK TKIs by activating by-pass compensatory signaling pathways in ALK-rearranged NSCLC [43, 46, 47] and are associated to EMT in different tumors types [48, 49]. Since we observed an up-regulation of both mRNA and protein levels of ERBB3 in TAE-684-inhibited H2228 cells (Figure 2B and Supplementary Figure 1A), we hypothesized an involvement of ERBB receptors in TAE-684 induced increased migration in H2228 cells. Indeed, treatment with lapatinib, a dual inhibitor of EGFR and ERBB receptors [50, 51], blocked the increased cell migration and invasion induced by TAE-684 (Supplementary Figure 5A-5B). We then further investigated the contribution of the EGFR family members to the EMT phenotype in ALK-rearranged NSCLC. To block EGFR signaling, we treated H2228 and DFCI032 with lapatinib, alone or in combination with ALK TKIs (TAE-684 or crizotinib). Lapatinib completely abrogated EGFR and ERBB2 signaling as detected by specific phospho-antibodies in both cell lines, but it did not affect E-cadherin or vimentin expression in contrast to ALK TKIs (Supplementary Figure 6A-6B). Thus, despite possible compensatory effects on the migratory and invasive abilities of ALK-rearranged cell lines, we concluded that EGFR and ERBB2 signaling did not significantly contribute to the EMT phenotype in ALK-rearranged NSCLC.

### EML4-ALK ectopic expression in immortalized lung epithelial cells induces a mesenchymal phenotype

**Figure 5:**
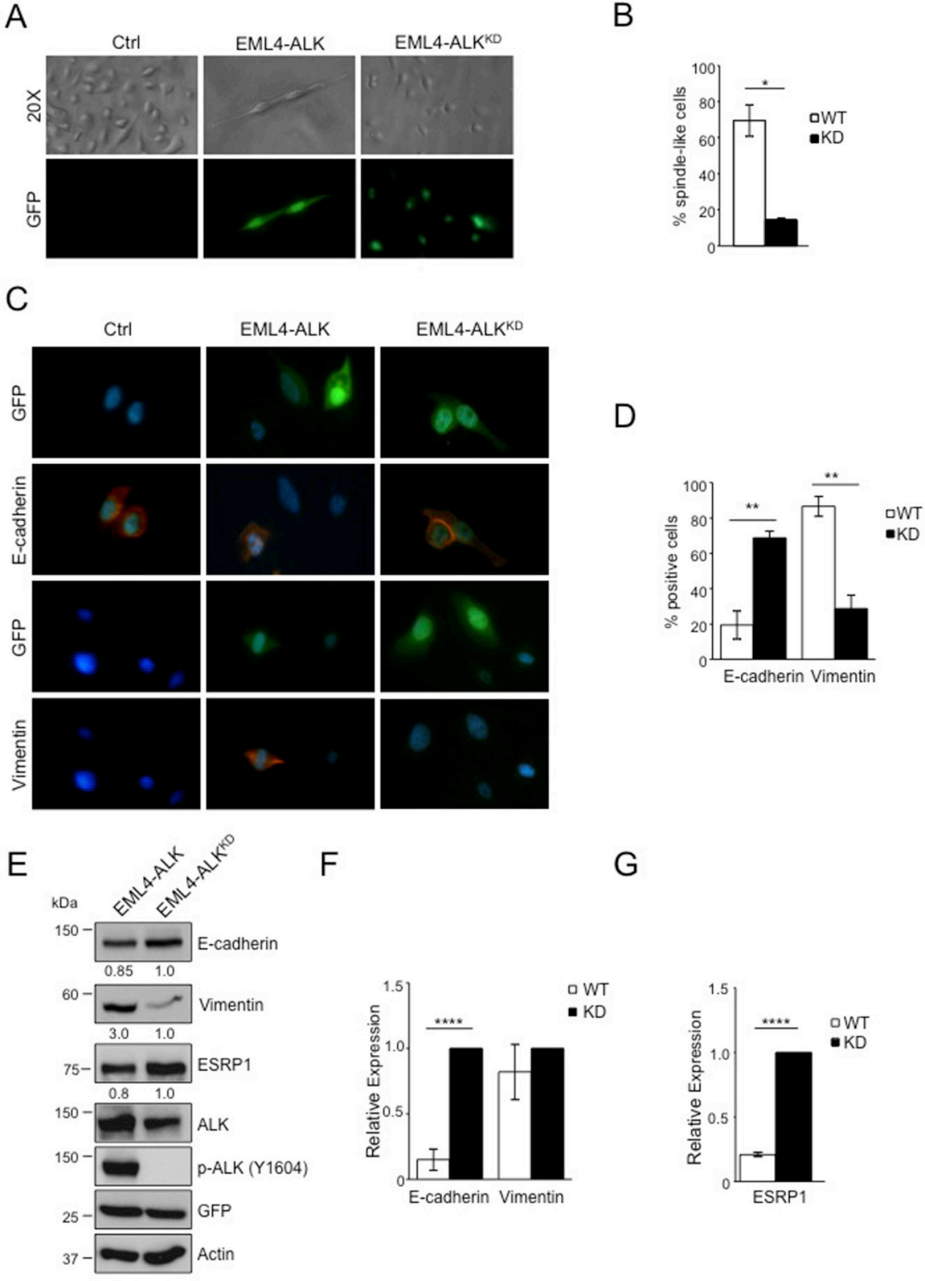
EML4-ALK ectopic expression in immortalized bronchial epithelial cells induces a mesenchymal phenotype BEAS-2B cell line were infected with GFP-retrovirus expressing either wild-type or kinase dead (KD) EML4-ALK. **A.** Cells are shown in phase contrast and under fluorescent detection to identify GFP-positive cells. **B.** Histogram displays the percentage of spindle-like cells over the total of GFP-reporter positive cells. **C.** Immunofluorescence staining for E-cadherin and vimentin (red fluorescence). Nuclei were stained with DAPI. **D.** Histogram shows the percentage of vimentin and E-cadherin positive cells over the total of GFP-reporter positive cells. One representative experiment out of two is shown. **E.** Cells were collected after infection and immunoblotted with the indicated antibodies. **F-G.** qRT-PCR analysis to detect mRNA levels of E-cadherin and vimentin (F) and ESRP1 (G). Two-tailed Student's t tests were used to calculate the p values shown. Data are represented as mean (±SEM). *, *P*<0.05; **, *P*<0.005; ****, *P*<0.0001.

Next, we asked whether EML4-ALK would promote an EMT phenotype also in non-tumoral lung cells. We stably transduced immortalized bronchial epithelial cells (BEAS-2B cells) with retroviruses expressing EML4-ALK or the kinase dead mutant K589R, EML4-ALK^KD^, as a control (Supplementary Figure 7A-7B). We observed dramatic morphological changes in BEAS-2B cells expressing EML4-ALK using light microscopy as the typically round-epithelial shape turned into an elongated spindle-shaped morphology in cells expressing EML4-ALK (Figure 5A, left and central panels) consistent with the acquisition of a mesenchymal-like morphology. Cells transduced with the kinase dead retained their epithelial morphology (Figure 5A, right panels). The percentage of spindle-shaped cells was significantly higher in cells expressing EML4-ALK compared to EML4-ALK^KD^ cells (Figure 5B). By immunofluorescence staining we found that enforced expression of EML4-ALK induced decreased E-cadherin and increased vimentin expression in GFP-reporter positive cells (Figure 5C-5D). In control cells transduced with EML4-ALK^KD^ both E-cadherin and vimentin expression and localization remained unaffected (Figure 5C-5D). Consistently, protein levels of E-cadherin and vimentin changed in presence of active EML4-ALK (Figure 5E), whereas only E-cadherin mRNA was regulated by EML-ALK in accordance with our findings in ALK-rearranged NSCLC cells (Figure 5F). Taken together, these findings demonstrate that oncogenic EML4-ALK not only maintains an EMT phenotype in NSCLC cells, but also induces a similar phenotype in normal epithelial lung cells.

### EML4-ALK regulates E-cadherin through repression of the epithelial splicing regulatory proteins 1 (ESRP1)

Finally, we investigated in more detail the mechanisms by which oncogenic ALK orchestrates the EMT phenotype in NSCLC. Our attention was caught by ESRP1 as it is considered to be a regulator of EMT by inducing a complex alternative splicing program [44]. ESRP1 induces E-cadherin expression and low levels of ESRP1 are typically associated with a mesenchymal phenotype in cancer cells [45, 52]. Therefore, because we showed that oncogenic ALK repressed the expression of ESRP1 in NSCLC (Figure 3 and Supplementary Figure 1B-1F and 2), we first confirmed that also in BEAS-2B cells the forced expression of EML4-ALK reduced ESRP1 levels, both mRNA and protein, in contrast to EML4-ALK^KD^ (Figure 5E and 5G), thus indicating that oncogenic ALK represses ESRP1 expression both in cancer and normal lung epithelial cells.

**Figure 6:**
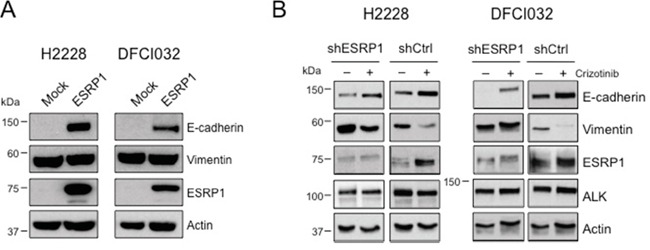
EML4-ALK regulates E-cadherin expression through ESRP1 repression **A.** H2228 and DFCI032 cell lines were transduced with a lentivirus expressing the human ESRP1, collected and blotted with the indicated antibodies. **B.** H2228 and DFCI032 were infected with pLKO expressing an shRNA targeting ESRP1 or a control shRNA (shCtrl). Cells were treated with ALK inhibitors for 96 hours and harvested for Western Blot analysis with the indicated antibodies.

Next we asked whether repression of ESRP1 is necessary for the maintenance of the EMT phenotype in ALK-rearranged cells. To test this hypothesis, we overexpressed ESRP1 in H2228 and DFCI032 cells. Enforced expression of ESRP1 alone produced a partial inversion of the EMT phenotype as it induced in both cell lines a marked expression of E-cadherin, but did not affect expression of vimentin (Figure 6A). Finally, in a reverse experiment, we asked whether ESRP1 up-regulation was required for the epithelial transition observed during ALK TKI treatment of NSCLC cells. To test this, we knocked-down ESRP1 by specific shRNA during crizotinib treatment and observed an attenuated E-cadherin induction compared to control shRNA in both cell lines (Figure 6B). Overall, these findings indicate that ESRP1 is a key regulator of the EMT phenotype induced by oncogenic ALK in normal and cancer lung epithelial cells.

## DISCUSSION

In the present study we show that the presence of ALK rearrangements correlated overall with an EMT molecular signature in NSCLC and that 2 out of 3 ALK-rearranged NSCLC cell lines displayed a mesenchymal phenotype, defined by the combined loss of epithelial markers, such as E-cadherin, and gain of mesenchymal markers, such as vimentin and N-cadherin. Importantly, we demonstrated that in those NSCLC with an EMT phenotype, EML4-ALK activity was necessary to sustain the phenotype by directly regulating the expression of proteins involved in EMT.

The association we found between EMT molecular signatures and ALK rearrangements in NSCLC is consistent with the frequent observation of the loss of E-cadherin associated with vimentin expression in ALK-rearranged lung cancer compared with other NSCLC genotypes [9]. In NSCLC driven by other oncogenes, such as EGFR or K-Ras, EMT is commonly associated with insensitivity to TKI treatment or loss of oncogene addiction [24, 25, 27]. In ALK-rearranged NSCLC previous studies reported the acquisition of an EMT phenotype associated with the acquired resistance to ALK TKI treatment, in some instances secondary to activation of TGF-β signaling pathway or induced by hypoxic conditions, in other cases by other still unknown mechanisms [26, 53–55]. None of the previous works, however, directly addressed the question whether sustained ALK oncogenic activity was responsible for the EMT phenotype in NSCLC. Here we describe that overall ALK-rearranged NSCLC cell lines and primary tumors intrinsically show an EMT signature, although with some degree of heterogeneity. Two cell lines, H2228 and DFCI032, showed an EMT phenotype, whereas H3122 cell line showed an epithelial differentiation completely lacking the expression of mesenchymal markers. The heterogeneous pattern of EMT is also a feature of primary lung cancers and cell lines driven by other oncogenes such as EGFR or K-Ras [20, 24, 27, 37], likely reflecting a multilayer complexity in the regulation of EMT in cancer [21]. Here, we provide evidence of a direct regulation of the EMT phenotype by EML4-ALK, a regulation not fully investigated in NSLCL driven by other oncogenic mutations such as EGFR and K-Ras [24, 27]. Indeed we showed that EGFR or ERBB family members did not contribute to the EMT phenotype in ALK-rearranged NSCLC. A recent report showed that EML4-ALK induced an EMT phenotype when overexpressed in an ALK-negative NSCLC cell line [30]. However, overexpression experiments are always difficult to interpret, given that the signaling of RTKs strictly depends on their levels of expression. Our study now provides evidence that oncogenic ALK regulates EMT at endogenous expression levels at least in a subset of NSCLC. In H2228 and DFCI032 cell lines the inhibition of ALK activity reverted the phenotype and caused the mesenchymal to epithelial transition (MET), down-regulating vimentin and concomitantly up-regulating E-cadherin. Because EMT is commonly associated with tumor progression and metastatization [15, 16] or resistance to therapy [24, 27-29, 53, 56], these findings have important implications for the biology and treatment of ALK-rearranged NSCLC. Interestingly, we and others previously showed that H2228 and DFCI032 are intrinsically less sensitive to ALK inhibition than the H3122 cell line [43, 57], thus raising the possibility that the sensitivity to ALK TKIs could depend on the EMT phenotype of each NSCLC determined by ALK itself. Moreover, we demonstrated that EML4-ALK directly promotes *in vitro* migration and invasion of NSCLC, thus suggesting that oncogenic ALK activity could contribute at least in part to the metastatic potential of NSCLC. To support this view, we previously showed that ALK controls *in vivo* liver metastasis formation in NSCLC xenografts in immunocompromised mice [58] and that the ALK oncogene directly regulates morphology, migration and cytoskeleton organization in ALK-rearranged lymphoma [59, 60]. Thus, the effects that ALK exerts on proteins related to cell morphology and motility seem to be a common feature of ALK-rearranged tumors independently from the cell or tissue origin. However, in particular in ALK-rearranged epithelial cells, the heterogeneity between tumors is high and several other molecules such as EGFR family members could contribute to the various phenotypes together with ALK.

EMT is mainly regulated by a complex network of transcription factors that potently induce the repression of E-cadherin and other junctional proteins, such as claudins and desmosomes [21, 22, 42]. These transcription factors, so called EMT-inducing transcription factors, belong to the Snail, Twist and Zeb families and are strongly interconnected to drive EMT in normal and cancer cells [22, 61]. We demonstrated that ALK activity directly regulated the protein expression levels of Snail, as previously reported [30], and ZEB1. In addition, EMT is also regulated post transcriptionally by alternative splicing of mRNA that encode specific protein isoforms related to the maintenance of the epithelial phenotype [22, 44, 52]. The epithelial splicing regulatory protein 1 and 2 (ESRP1/2), master regulators of EMT, are epithelial-specific proteins that maintain a specific splicing program in epithelial cells. Repression or low expression of ESRP1/2 causes a splicing switch that induces a mesenchymal phenotype and control E-cadherin expression [52]. We found that in H2228 and DFCI032 cell lines ESRP1/2 were expressed at low levels. Importantly, in both normal and lung cancer cells ESRP1/2 were regulated by ALK oncogenic activity. ESRP1 repression resulted in E-cadherin down-regulation and ESRP1 enforced expression led to E-cadherin up-regulation in ALK-rearranged NSCLC. These findings suggest that ALK sustains the mesenchymal phenotype in NSCLC by inducing a transcriptional regulation of E-cadherin and an alternative splicing program mediated by ESRP proteins.

Our findings might have important clinical implications because an EMT phenotype could contribute to predict TKI response and metastatic potential in each ALK-rearranged NSCLC. In this context, our findings that in NSCLC with an EMT phenotype, the inhibition of ALK activity by TKIs at least partially reverts the phenotype could have implication in the design of novel therapeutic strategies that rely on different drug combinations.

## MATERIALS AND METHODS

### Cell lines and reagents

Human ALK-rearranged NSCLC cell lines, H2228 (variant 3, E6;A20), DFCI032 and H3122 (variant 1, E13;A20), were grown in DMEM (Lonza) with 10% FCS (Lonza). 293T packaging cells, were cultured in DMEM (Lonza) with 10% FCS (Lonza). The H2228 TTA A5 (shALK) were generated as previously described and shRNA expression wad induced with 1μg/ml doxycycline-hyciclate (Sigma) [43]. BEAS-2B cells were purchased from ATCC (# CRL9609) and grown in bronchiolar epithelial cell basal medium (Lonza; #CC-3170).

For ALK inhibitors, NVP-TAE684 was purchased from Axon Medchem and Crizotinib (PF-02341066) was kindly gifted by Pfizer.

### DNA constructs, virus preparation and cell infection

The retroviral vector, pallino, expressing EML4-ALK or the kinase dead, EML4-ALK^K589R^ was previously described [43].

Lentiviral vectors containing shRNA targeting ESRP1 or a control sequence were obtained from Sigma. Lentiviral vector containing ESRP1 cDNA was previously described [62]. Retroviruses and lentiviruses were generated and cells were infected as previously described [43].

### Western blot analysis

Cells were lysed as previously described [43]. The following antibodies were used: anti-ALK (clone D5F3), anti-phospho-ALK (Y1604), anti-E-cadherin, anti-vimentin, anti-N-cadherin, anti-phospho-EGFR (Y1068), anti-EGFR (clone D38B1), anti-phospho-ERBB2 (Y1221-Y1222), anti-ERBB2 (clone D8F12), anti-ERBB3 (clone D22C5) and anti-Snail, were from Cell Signaling Technology; anti-ZEB1 (clone H-102) and anti-Twist (clone H-81) were from Santa Cruz Biotechnology; anti-PTP4A1 was from Proteintech; anti-ESRP1/2 was from Rockland and anti-actin was from Sigma.

### Data mining on public available ALK-rearranged NSCLC

Eleven ALK positive samples were collected from GEO31210, 5 annotated ALK fusion samples from TCGA Lung Adenocarcinoma Dataset (LUAD), and 29 adjacent normal samples from TCGA LUAD.

Gene normalization was used to normalize all GEO data based on all TCGA cancer samples in order to remove batch effect and technology bias. KS test was applied in R to test whether EMT signatures (n=6) in MSigDB were enriched in the differentially expressed genes between ALK-rearranged tumors and normal tissues [35]. To test whether EMT signatures can distinguish normal and tumor samples, hierarchical clustering was performed on an EMT activated signatures (“ALONSO_METASTASIS_UP”) of normalized expression levels both on the gene and sample levels.

### RNA sequence analysis

100nt Paired-end RNA-Seq was performed on H2888 cells treated with TAE-684 for 48 and 72 hours. Untreated H2228 were used as control. The reads were aligned to hg19 reference using Tophat aligner [38]. Cuffdiff was used to identify differentially expressed genes in cells treated with ALK inhibitor (TAE-684) and cells treated with DMSO (controls) (FDR 0.05 cutoff) [38]. Hyper-geometric test was performed on both the up-regulated and down-regulated genes upon ALK inhibition against MSigDB C2 CGP signature sets [35].

### RT^2^ profiler PCR array

Real-time PCR was used for RT^2^ Profiler PCR Array (96-well format) for Human Epithelial to Mesenchymal Transition (EMT) (QIAGEN) in combination with IQ Sybr Green Supermix (BIO-RAD) according to manufacturer's instructions. RT-PCR was carried out on a BIO-RAD iCycler and calculation of the threshold cycles was performed using iCycler. Definition of the baseline, the threshold and statistical analysis were obtained according to the manufacturer's instructions. Normalization was performed against normalization genes provided by array plate.

### Immunofluorescence

Adherent cells were grown on glass coverslips, fixed in 4% para-formaldehyde and incubated with the primary antibodies (same as Western Blot) as previously described [59]. Nuclei were counterstained with DAPI. Cells were evaluated with a 40x objective using a Leica photomicroscope and images were acquired using the DM LM Leica software.

### Histology and immunohistochemistry

For histological evaluation, tissue samples were fixed in formalin, embedded in paraffin, stained and visualized as previously described [58]. Paraffin-embedded tissue sections (2-μm thick) were immunostained with primary antibodies for E-cadherin, vimentin and ESRP1 on a semiautomated immunostainer. Anti-E-cadherin and anti-vimentin antibodies were the same used for Western blot analysis. For IHC, anti-ESRP1 was from ThermoFisher.

### Quantitative real time PCR (qRT-PCR)

Total RNA from cell lines was extracted from cells using Trizol (Ambion Invitrogen) according to the manufacturer's instructions. Reverse transcription–polymerase chain reactions (RT-PCRs) were performed from 1μg RNA using random primers and SuperScript II Reverse Transcriptase (Invitrogen). Real-time RT-PCR was carried out on a BIO-RAD iCycler using IQ Sybr Green Supermix (BIO-RAD). Calculation of the threshold cycles was performed using iCycler and normalization was performed against HUPO housekeeping gene according to the formula 2-ΔΔCt where ΔCt = Ct (threshold cycle) gene of interest − Ct internal control, as indicated by the manufacturer.

Primers used in quantitative RT-PCR for:

**Table:** 

Gene	Forward	Reverse
E-cadherin	CGGGAATGCAGTTGAGGATC	AGGATGGTGTAAGCGATGGC
Vimentin	CTCTTCCAAACTTTTCCTCCC	AGTTTCGTTGATAACCTGTCC
PTP4A1 (PRL-1)	ACCTGGTTGTTGTATTGCTGTT	GTTGTTTCTATGACCGTTGGAA
SerpinE1	ACCTCTGAGAACTTCAGGATGC	ACCTGCTGAAACACCCTCAC
HUPO	GCTTCCTGGAGGGTGTCC	GGACTCGTTTGTACCCGTTG

### Migration and invasion assays

Migration assays were performed using Transwell^®^ Permeable Supports (0,8μm pores; 24 well multiple well plate; Corning). Invasion assays were performed using BD BioCoat Matrigel Invasion Chambers (BD Biosciences). Cells were serum starved 24 hours prior to migration assay. 5 × 10^4^ cells/well in 100μl serum-free media containing DMSO or 300nM ALK TKIs, TAE-684 or crizotinib, were placed in the upper chamber of the transwell and then placed in 24-well plate with DMEM containing 20% FCS as a chemoattractant. Non-migrated cells were removed from the top of the inserts with a cotton swab and migrated cells were fixed and colored with Giemsa. Migrated cells were counted using a light microscope. The number of migrated cells was evaluated on five fields per chamber (20x objective). The percentage of migration or invasion was calculated relative to the controls treated with DMSO.

### Mice and *in vivo* experiments

NOD-SCID mice (Charles River Laboratories Italia S.p.A) were inoculated s.c in both flanks (single cell suspension, 1 × 10^7^ cells in 0.2 ml PBS). Four mice were included in the control group (treated with vehicle) and 4 mice in the treatment group. Treatment started when s.c. tumors reached 0.5 cm mean diameter. Mice were treated once a day with 25mg/kg of TAE-684. Mice were sacrificed after 5 days of inhibitor treatment to perform histology and immunohistochemistry. Mice were handled and treated in accordance with European Community guidelines.

### Statistical analysis

Statistical significance was calculated with T-Student test. P values of <0.05 were considered significant. Unless otherwise noted, data are presented as means ± sd.

## SUPPLEMENTARY FIGURES AND TABLES






