# Supplementary material for: Oncogenic ALK regulates EMT in non-small cell lung carcinoma through repression of the epithelial splicing regulatory protein 1
Source: Oncotarget. 2016 Apr 23;7(22):33316–30. doi: 10.18632/oncotarget.8955 (PMC5078097; doi:10.18632/oncotarget.8955)
Supplement: Supplementary file 1 [file oncotarget-07-33316-s001.pdf]

SUPPLEMENTARY FIGURES AND TABLES

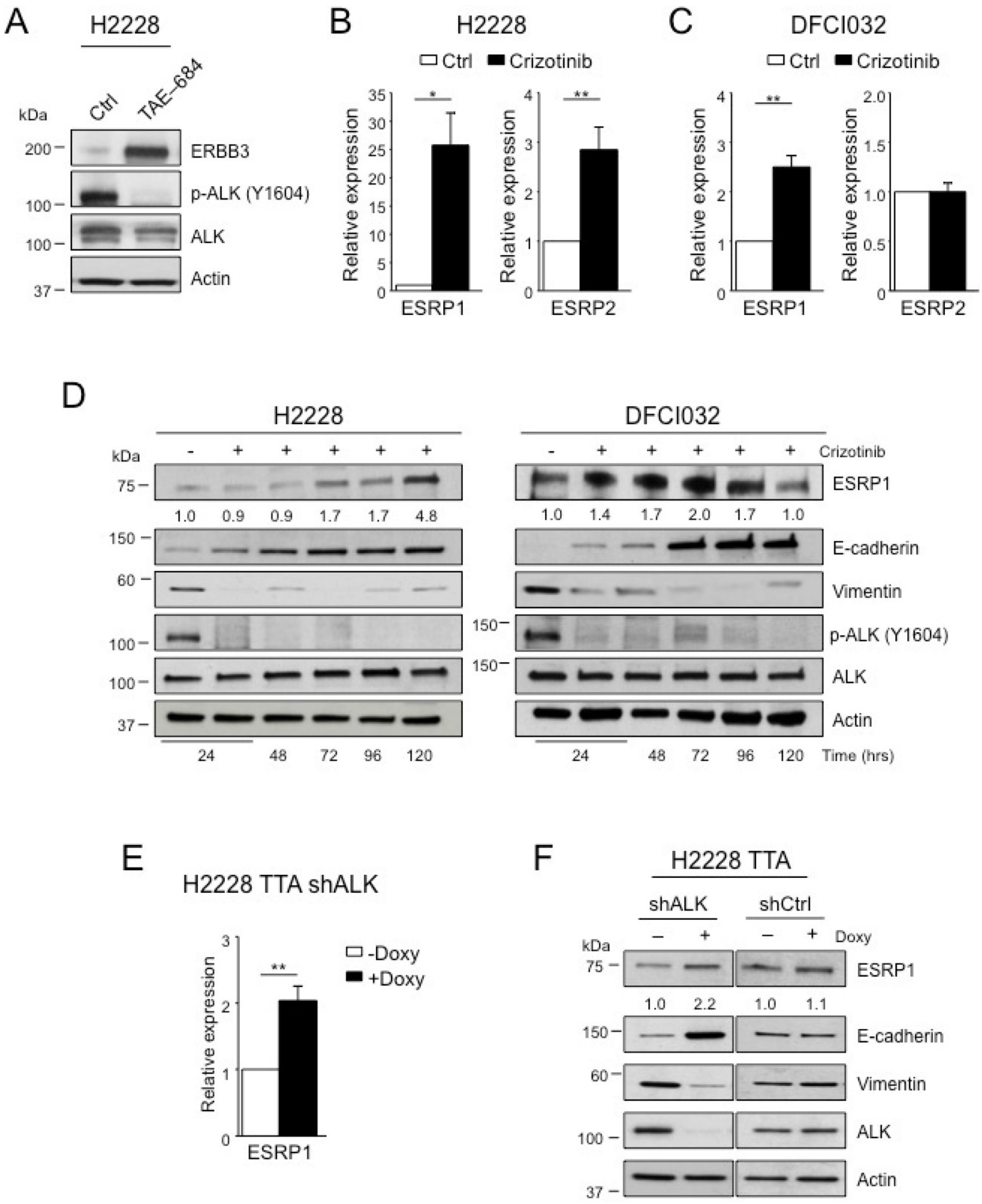

Supplementary Figure S1: EML4-ALK transcriptionally regulates ESRP1 and 2. A. H2228 and DFCI032 cell lines were treated for 24 hours with 300nM TAE-684. Total cell lysates were blotted with the indicated antibodies. **B-C.** H2228 (B) and DFCI032 (C) were treated with crizotinib (150nM) for 96 hours and collected for qRT-PCR analysis to check mRNA expression of ESRP1 and ESRP2. One representative experiment out of two is shown. **D.** H2228 and DFCI032 cell lines were treated with crizotinib (300nM) for the indicated time. Cells were collected and blotted with the indicated antibodies. **E-F.** The doxycycline inducible H2228 TTA cell line was stably transduced with a shRNA targeting ALK (shALK) or a control shRNA (shCtrl). Cells were collected at 120 hours of induction with doxycycline for qRT-PCR analysis of ESRP1 mRNA (E) and Western blot analysis with the indicated antibodies (F). Two-tailed Student's t tests were used to calculate the p values shown. Data are represented as mean (±SEM). *, $P<0.05$; **, $P<0.005$.

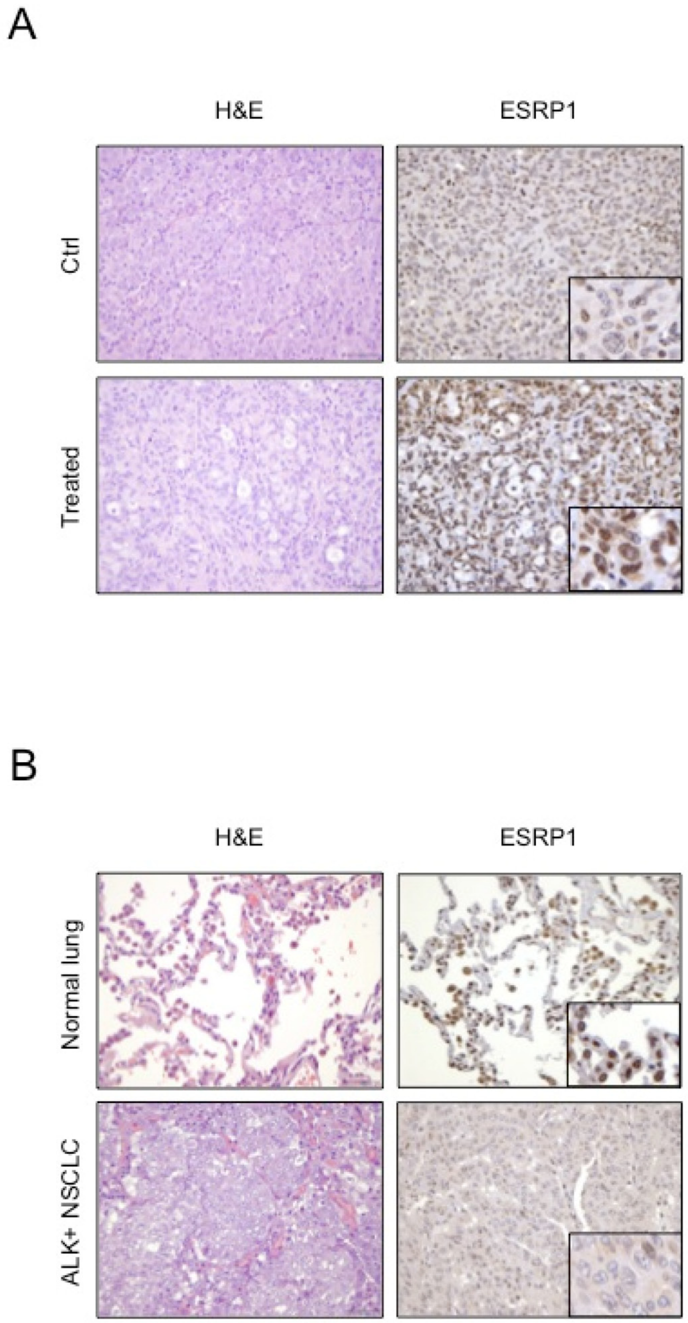

Supplementary Figure S2: ALK represses the expression of ESRP1 in ALK-rearranged NSCLC. A. Representative hematoxilin-eosin (H&E) (left panels) and immunostaining with anti-ESRP1 (right panels) on tumor xenograft sections from control (Ctrl) and mice treated with TAE-684. Data are from two independent experiments. **B.** Representative hematoxilin-eosin (H&E) (left panels) and immunostaining with anti-ESRP1 (right panels) on a section of primary lung tumor (lower panels) and the adjacent normal lung (upper panels) of ALK+ NSCLC case.

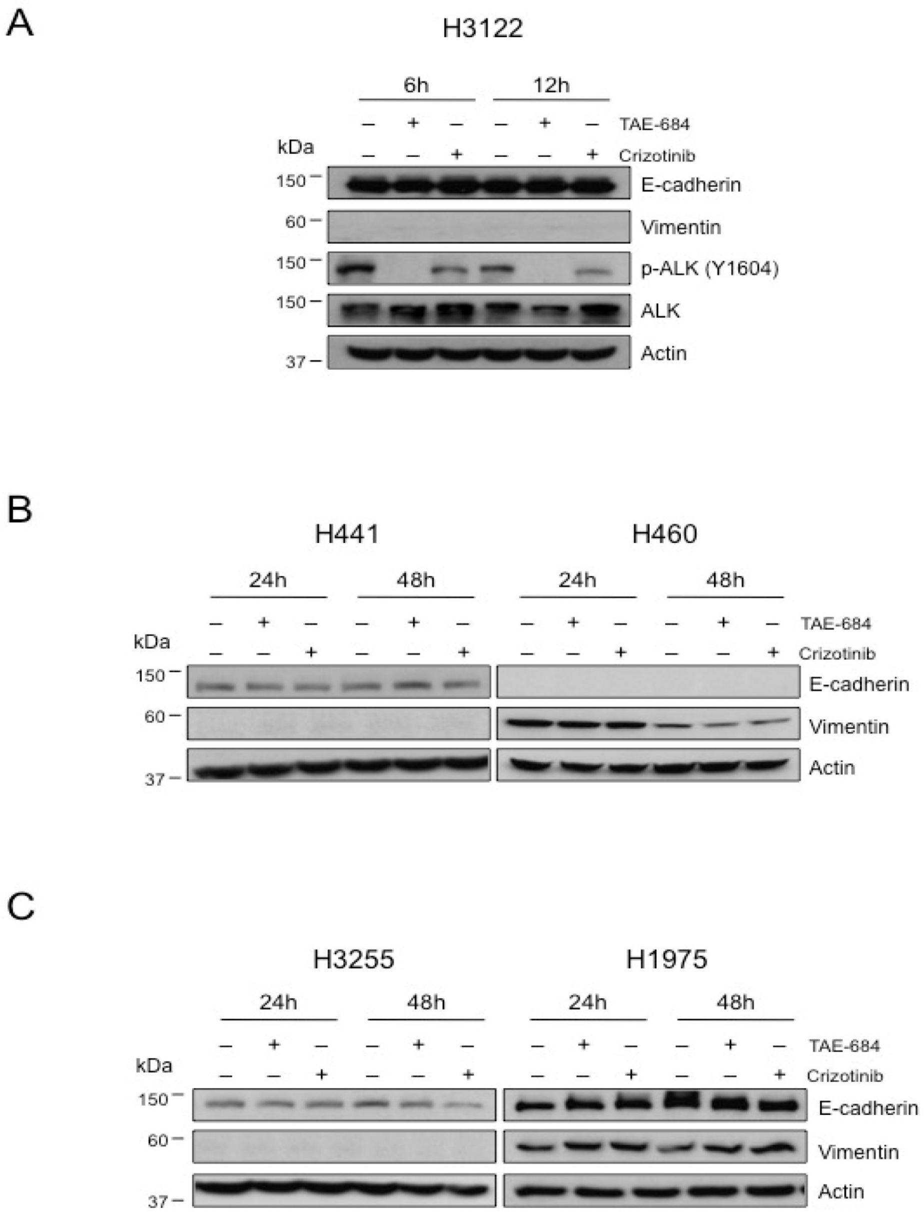

Supplementary Figure S3: ALK TKIs do not affect EMT markers in NSCLC with EGFR or K-Ras mutations. A. H3122 cell line was treated with TAE-684 or crizotinib (300nM) for 6 and 12 hours. Total cell lysates were blotted with the indicated antibodies. **B-C.** K-Ras-mutated lung cancer cell lines (H441 and H460) (B) and EGFR-mutated NSCLC cells (H3255 and H1975) (C) were treated with TAE-684 or crizotinib (300nM) for 24 and 48 hours. Total cell lysates were blotted with the indicated antibodies.

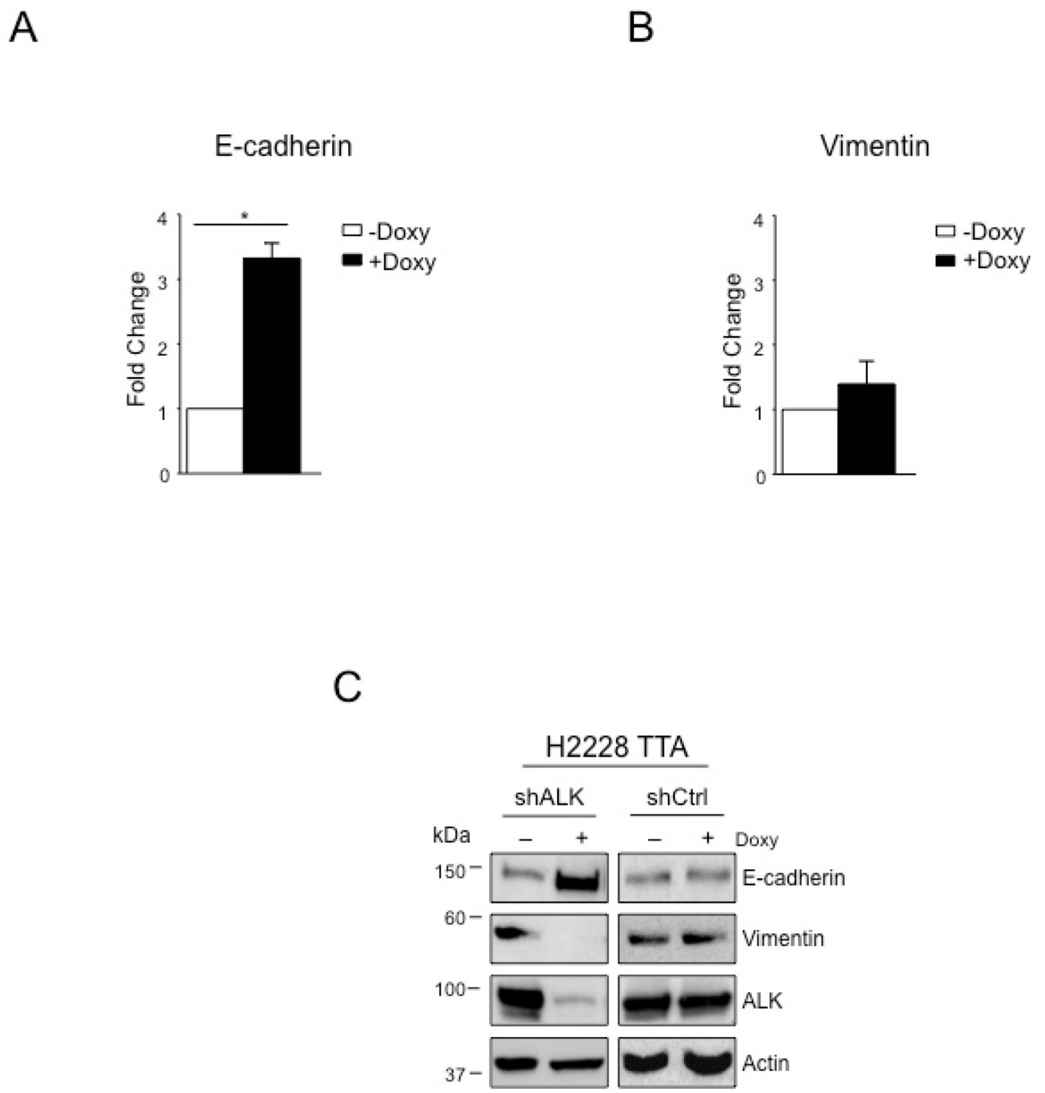

Supplementary Figure S4: EML4-ALK knock-down by shRNA induces the mesenchymal-epithelial transition (MET). **A-B.** The doxycycline inducible H2228 TTA cell line was stably transduced with a shRNA targeting ALK (shALK) or a control shRNA (shCtrl). Cells were treated with 1µg/mL doxycycline for 96 hours. qRT-PCR analysis was performed to check mRNA levels of E-cadherin (A) and vimentin (B). **C.** H2228 TTA A5 (shALK) cells were treated with 1µg/mL doxycycline for 96 hours and collected. Total cell lysates were blotted with the indicated antibodies. Two-tailed Student's t test was used to calculate the p values shown. All data are presented as the mean (±SEM). For E-cadherin: *, $P<0.05$. For vimentin the p value was not significant.

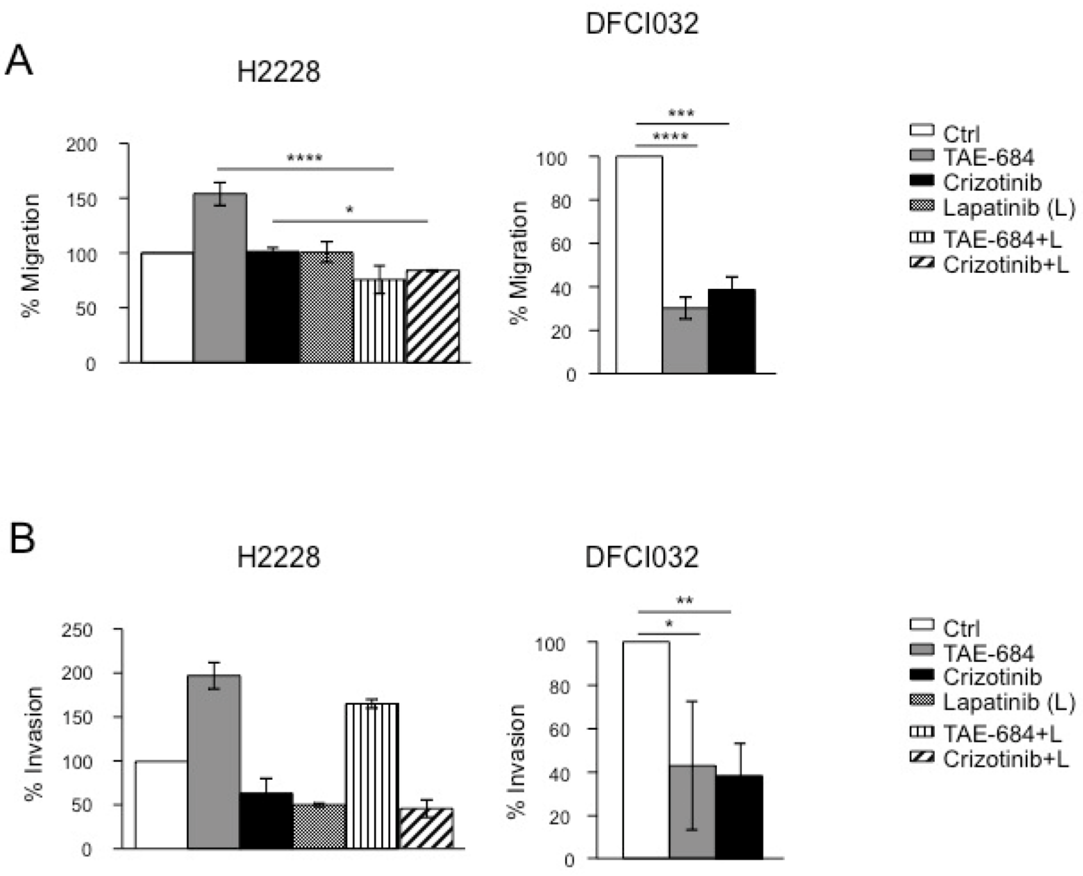

Supplementary Figure S5: EML4-ALK inhibition affects cell migration and invasion of DFCI032 cells. A-B. Percentages of migration (A) and invasion (B) relative to controls at 12 hours and 24 hours, respectively, in H2228 and DFCI032 cell lines are shown. Histograms represent one experiment out of three. Two-tailed Student's t tests were used to calculate the p values shown. Data are represented as mean (±SEM). *, $P<0.05$; **, $P<0.005$; ***, $P<0.0005$; ****, $P<0.0001$.

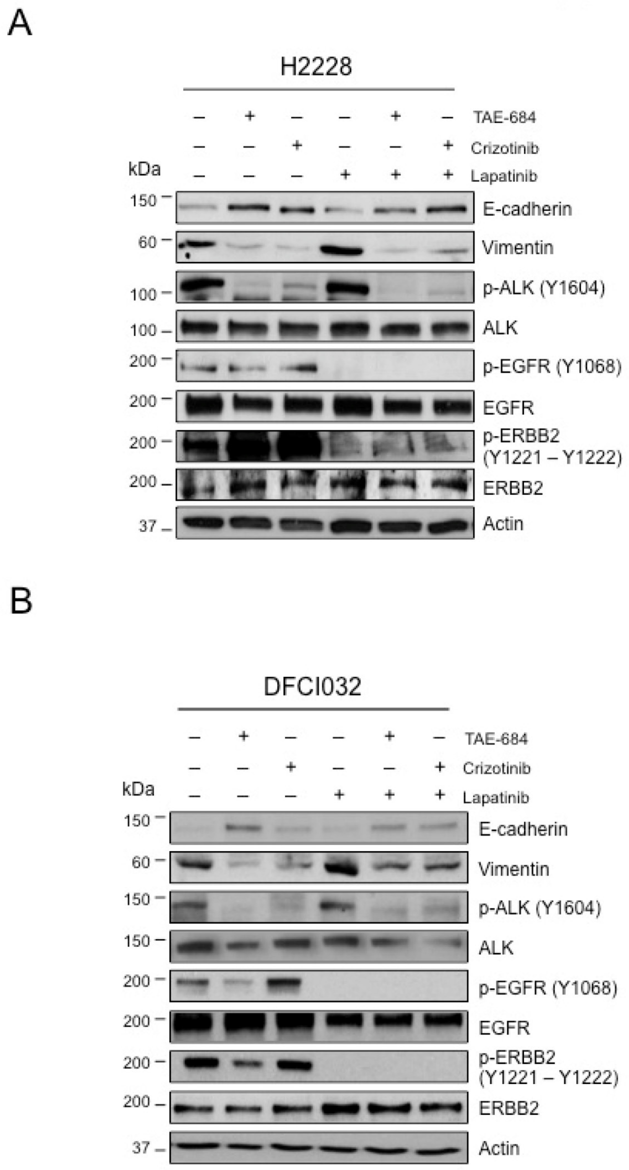

Supplementary Figure S6: EGFR family members do not contribute to the EMT phenotype in ALK-rearranged NSCLC cells. A-B. H2228 (A) and DFCI032 (B) cells were treated with 300nM TAE-684 or crizotinib or 1µM lapatinib in single or in combination for 48 hours. Total cell lysates were blotted with the indicated antibodies.

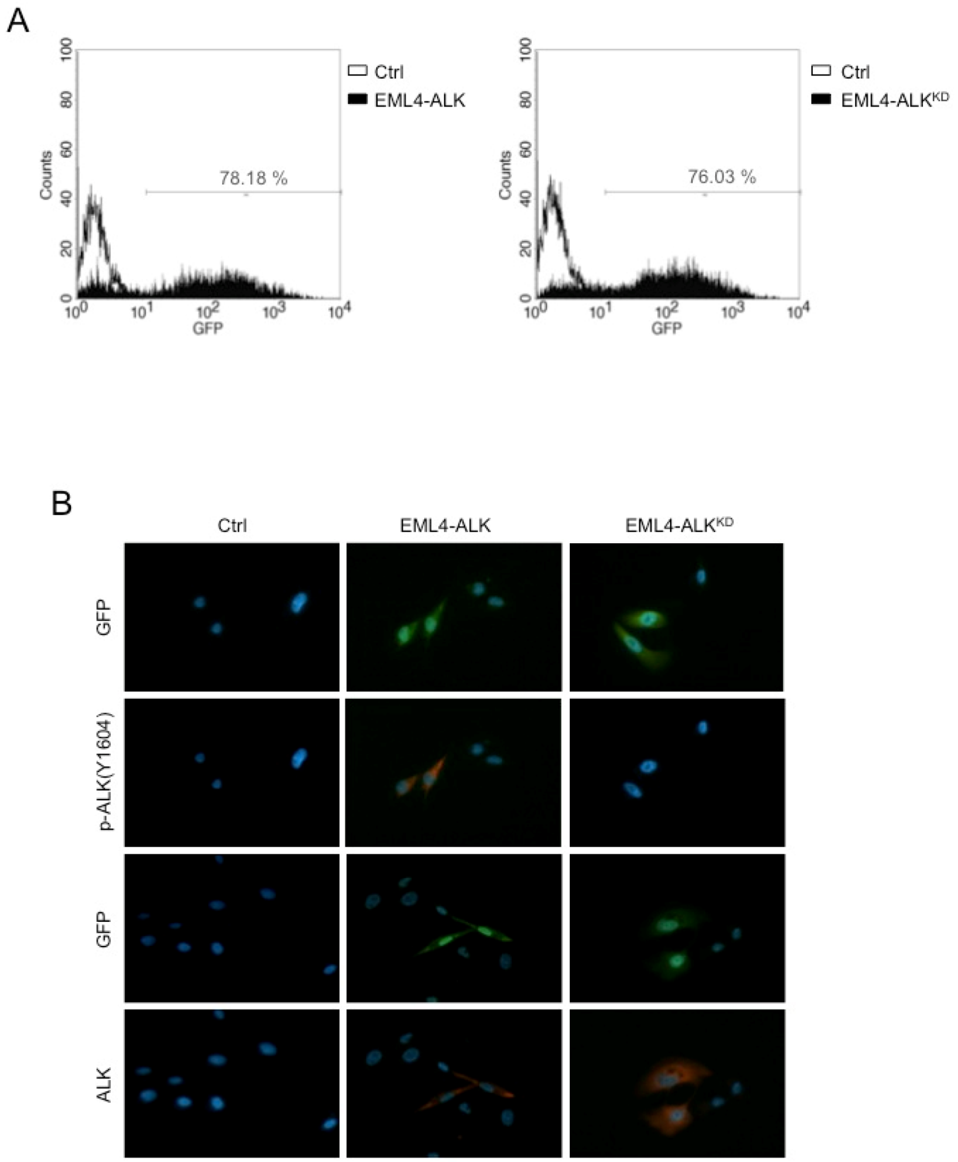

Supplementary Figure S7: A. BEAS-2B cells were infected with retroviruses expressing EML4-ALK (variant 1) and the kinase dead mutant K589R (EML4-ALK^{KD}). The percentage of infected GFP-reporter positive cells was analyzed by flow-cytometry at 72 hours from infection. **B.** Immunofluorescence staining with an anti-ALK or anti-pALK (Y1604) antibodies (red fluorescence). Green fluorescence is the GFP-reporter protein encoded by transduced cells. Nuclei were stained with DAPI.

Supplementary Table S1: Down-regulated and up-regulated genes upon ALK inhbition with TAE-684 in H2228 cell line by RNA-seq analysis

See Supplementary File 1

Supplementary Table S2: List of genes in EMT RT2 Profiler PCR Array

See Supplementary File 2
